# Long-term study of the efficacy and safety of OnabotulinumtoxinA for the prevention of chronic migraine: COMPEL study

**DOI:** 10.1186/s10194-018-0840-8

**Published:** 2018-02-05

**Authors:** Andrew M. Blumenfeld, Richard J. Stark, Marshall C. Freeman, Amelia Orejudos, Aubrey Manack Adams

**Affiliations:** 1 Headache Center of Southern California, The Neurology Center, 6010 Hidden Valley Road, Carlsbad, CA 92024 USA; 2 Monash University and Alfred Hospital, Melbourne, VIC Australia; 3 Headache Wellness Center, Greensboro, NC USA; 4 Allergan plc, Irvine, CA USA

**Keywords:** OnabotulinumtoxinA, Efficacy, Safety, Long-term, Chronic migraine, Prophylaxis

## Abstract

**Background:**

OnabotulinumtoxinA is approved for the prevention of headache in those with chronic migraine (CM); however, more clinical data on the risk-benefit profile for treatment beyond one year is desirable.

**Methods:**

The Chronic Migraine OnabotulinuMtoxinA Prolonged Efficacy open Label (COMPEL) Study (ClinicalTrials.gov, NCT01516892) is an international, multicenter, open-label long-term prospective study. Adults with CM received 155 U of onabotulinumtoxinA (31 sites in a fixed-site, fixed-dose paradigm across 7 head/neck muscles) every 12 weeks (±7 days) for 9 treatment cycles (108 weeks). The primary outcome was headache day reductions at 108 weeks; secondary outcomes were headache day reductions at 60 weeks and change in the 6-item Headache Impact Test (HIT-6) score. Safety and tolerability were assessed by reviewing the frequency and nature of adverse events (AEs). AEs were determined at each visit through patient self-report, general non-directed and, for specific AEs, directed questioning, and physical examination. Subgroup analyses for safety and efficacy included, but were not limited to, patients with/without concomitant oral preventive treatment and acute medication overuse at baseline.

**Results:**

Enrolled patients (*N* = 716) were 18–73 years old and most were female (*n* = 607, 84.8%). At baseline, patients reported an average 22.0 (SD = 4.8) headache days per month. 52.1% of patients (*n* = 373) completed the study. By 60 and 108 weeks, a significant reduction in headache days (− 9.2 days and − 10.7 days, respectively, *P* < 0.0001) was observed. Significant improvements (*P* < 0.0001) in HIT-6 scores (− 7.1 point change at week 108) were also demonstrated. 131 patients (18.3%) reported ≥1 treatment-emergent adverse events; most frequently reported was neck pain (*n* = 29, 4.1%). One patient reported a serious treatment-related adverse event (rash). No deaths were reported.

**Conclusions:**

The COMPEL Study provides additional clinical evidence for the consistency of the efficacy and for the long-term safety and tolerability of onabotulinumtoxinA for the prevention of headache in those with CM who have been treated with onabotulinumtoxinA every 12 weeks over 2 years (9 treatments) with the fixed-site, fixed-dose injection paradigm.

**Trial registration:**

Trial registration number: NCT01516892. Name of registry: clinicaltrials.gov. Date of registration: January 20 2012. Date of enrollment of first patient: December 2011.

**Electronic supplementary material:**

The online version of this article (10.1186/s10194-018-0840-8) contains supplementary material, which is available to authorized users.

## Background

Chronic migraine (CM) is a debilitating neurologic disease defined as headaches that occur on ≥15 days per month for > 3 months, with headaches having migraine features on ≥8 days per month [1]. CM affects approximately 1.4% to 2.2% of adults worldwide [2, 3] and has a substantial quality of life (QoL) and economic burden [4–9]. Both the frequency of attacks and the severity of the pain and associated symptoms have an impact on migraine-related disability [4]. Individuals with CM experience substantially greater headache-related disability [4, 10–12] than individuals with episodic migraine (EM).

High levels of headache-related disability reflect an unmet treatment need [4]. Despite the impact on QoL, < 50% of those with CM consistently take preventive medications [10, 13]. This is supported by large population-based longitudinal surveys which show that many people with CM do not receive adequate migraine treatment [13, 14].

OnabotulinumtoxinA is approved for prevention of headache in adults with CM. The Phase III REsearch Evaluating Migraine Prophylaxis Therapy (PREEMPT) clinical trials established the safety and efficacy of onabotulinumtoxinA for the treatment of CM [15–17]. OnabotulinumtoxinA reduced the frequency of headache days and of moderate or severe headache days and significantly improved health-related QoL at the end of the 24-week double-blind treatment period compared with placebo, [16] with a further reduction in the frequencies of headache days and moderate or severe headache days and improvement in health-related QoL at the end of the 32-week open-label phase (56-week total treatment period) [17].

The Chronic migraine OnabotulinuMtoxinA Prolonged Efficacy open Label (COMPEL; NCT01516892) Study was designed to expand on the current 56-week efficacy and safety data by evaluating the long-term efficacy and safety of onabotulinumtoxinA for prevention of headache in those with CM. Due to the extended duration of the study (108 weeks), and the established efficacy and safety of onabotulinumtoxinA in people with CM at 1 year, an open-label study was considered the optimal design. A randomized controlled trial would have led to a long period of exposure to placebo and possibly excessive discontinuation rates. It is likely that such an approach would not have been accepted by ethics committees. Alternatively, an observational study design and real-world data can be used to extend our knowledge of the safety and effectiveness profile of onabotulinumtoxinA when used in clinical practice. However, given that physicians often do not treat per label in practice, an observational design would not have evaluated the efficacy of onabotulinumtoxinA when used every 12 weeks with a fixed-site, fixed-dose injection paradigm, which was the primary research question evaluated by the COMPEL Study. Both real-world and open-label study data have considerable clinical utility because the combination of data from such studies can help inform physicians on how to use onabotulinumtoxinA to optimally manage people with CM. The COMPEL Study also sought to evaluate outcomes in addition to the those related to reduction in headache days, including the effect of treatment on related comorbidities and quality-of-life measures. Herein, we report the primary and secondary outcomes for the COMPEL Study, as well as the subgroup analyses for these outcomes.

## Methods

### Study design

**Fig. 1:**
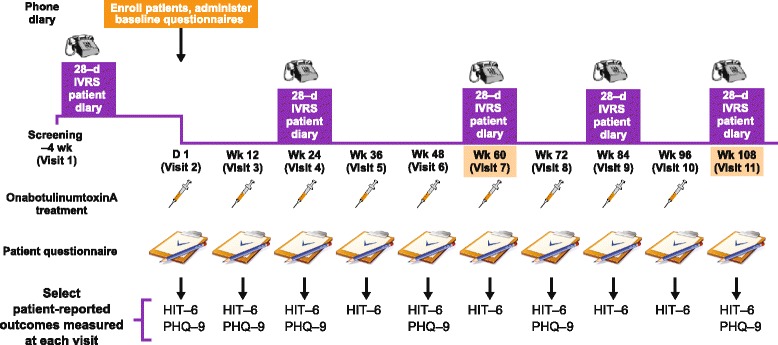
Overview of COMPEL Study design. HIT-6 = 6-item Headache Impact Test; IVRS = interaction voice response system; PHQ-9 = 9-item Patient Health Questionnaire. Adapted from Blumenfeld AM, Aurora SK, Laranjo K, Papapetropoulos S. Unmet Clinical Needs in Chronic Migraine: Rationale for Study and Design of COMPEL, an Open-Label, Multicenter Study of the Long-Term Efficacy, Safety, and Tolerability of OnabotulinumtoxinA for Headache Prophylaxis in Adults With Chronic Migraine. *BMC Neurol.* 2015; 15: 1–9

The COMPEL Study (clinicaltrials.gov identifier NCT01516892) was an international, multicenter, open-label long-term prospective study in adults with CM at 35 sites in the United States (*n* = 24), Australia (*n* = 5), and Korea (*n* = 6). The enrollment period was December 2011 to October 2013. The study design has been previously published [18]. The study duration was 112 weeks, including a 4-week baseline period and a 108-week, open-label treatment intervention phase (Fig. 1).

Demographics, medical history, physical exam, headache features, and headache treatment history were recorded at the baseline visit (week 0). Diary data (entered via interactive voice response system [IVRS]) for efficacy assessment were captured by the patient for the 28 days before the baseline visit, and then for the 28 days before week 24 (after treatment 2), week 60 (after treatment 5), week 84 (after treatment 7), and week 108 (after treatment 9).

OnabotulinumtoxinA (BOTOX®; Allergan plc, Dublin, Ireland) 155 U was administered every 12 weeks using the US Food and Drug Administration approved fixed-site, fixed-dose injection paradigm into 7 muscle areas and 31 sites [19].

The study received ethical approval from the Institutional Review Board or Independent Ethics Committee at each site, and written informed consent was obtained from patients before study enrollment.

### Study participants

Adults aged ≥18 years with a diagnosis of CM, able to follow the study instructions, attend the treatment and follow-up visits, and with stable comorbidities were eligible for study inclusion. Physicians working in headache centers or tertiary institutions were responsible for ensuring patients met the criteria for CM, including having a diagnosis of migraine headache disease with headaches on ≥15 days per month lasting ≥4 h a day. Patients could take a single oral medication as headache prevention. The dose and regimen of the oral preventive treatment must have been stable for > 4 weeks before the first intervention visit (week 0, visit 2). The dose could then not be changed until at or after week 24. If a patient was not on any oral preventive treatment at week 0, they must not have been on oral preventive treatment for the preceding 4 weeks and could only have an oral preventive treatment added after week 24.

Patients could take acute headache medication on an as-needed basis and were required to record the use of acute headache medication in their daily patient diary. At baseline, patients were defined as overusing acute headache medication if they were taking acute headache medication ≥2 times a week in any week with diary data on ≥5 days for the 4-week screening period. This differs from the definition of medication overuse adopted by the International Headache Society, which requires 3 months of medication overuse and has drug-specific treatment day minima [1].

Patients were excluded if they had previously received onabotulinumtoxinA for any reason, did not meet the study criteria for CM or had severe major depressive disorder or suicidal ideation [18].

### Efficacy outcome measures

As recommended by the International Headache Society Clinical Trials Subcommittee Guidelines, [20] the primary efficacy measure was the number of headache days per 28-day period (headache frequency) immediately before week 108. Efficacy measures were based on daily diaries (recorded via IVRS).

Secondary efficacy measures included headache frequency at week 60, and change in 6-item Headache Impact Test (HIT-6) scores from baseline at weeks 60 and 108. HIT-6 is a 6-domain patient survey used to assess the impact of headaches. Each of the 6 questions was scored and summed for a total possible score of 36 to 78, with higher scores indicating a greater adverse impact [21].

Exploratory efficacy measures included reduction in frequency of moderate or severe headache days.

A headache day was a day (00:00 to 23:59) for which the patient recorded ≥4 continuous hours of headache. Patients rated all headaches on a 4-point scale: 0 = none, 1 = mild, 2 = moderate, 3 = severe. A moderate or severe headache day was defined as a day with ≥4 continuous hours of headache that the patient had rated as moderate or severe [21].

#### Subgroup analysis

Subgroup analysis was undertaken based on race (Caucasian vs non-Caucasian), comorbid anxiety (none vs mild or moderate defined by Generalized Anxiety Disorder-7 score), comorbid depression (none vs mild or moderate defined by Patient Health Questionnaire-9 total score), body mass index (< 18.5 kg/m^2^ vs 18.5 to < 25 kg/m^2^ vs 25 to < 30 kg/m^2^ vs ≥ 30 kg/m^2^), history of acute headache medication overuse (yes vs no), age (18 to < 25 years vs 25 to ≤65 years vs > 65 years), use of oral preventive treatment for headache at baseline (yes vs no), previous use of preventive treatment for headache (yes vs no), and country (United States, Australia, and South Korea).

### Safety and tolerability

Safety and tolerability were assessed by reviewing the frequency and nature of adverse events (AEs). AEs were determined at each visit through patient self-report, general non-directed questioning, direct questioning via the Columbia-Suicide Severity Rating Scale, and physical examinations. AEs were recorded starting at week 0 immediately after the first onabotulinumtoxinA treatment.

Patients were withdrawn from the study for safety reasons if they showed any signs of suicidal ideation or if they became pregnant [18]. They received no further protocol-related onabotulinumtoxinA treatment; however, these patients were included in the safety and tolerability analysis.

### Statistical analysis

Based on the efficacy of onabotulinumtoxinA in the PREEMPT studies, assuming a standard deviation of 6.6, a sample size of 60 patients was considered sufficient to provide at least 80% power to detect between subgroup differences of ≥2.5 headache days reduction per 28-day period with a 95% significance level. Assuming that a subgroup was approximately 10% of the analysis population, an overall sample size of 600 patients was required to detect subgroup differences as outlined above. Subgroup analysis was only performed if it had sufficient individuals (*n* ≥ 60) to detect significant differences.

For the primary efficacy endpoint, an intention-to-treat analysis was undertaken on all patients with ≥1 efficacy assessment. Missing headache days data were imputed using a modified last observation carried forward (mLOCF) methodology, with imputation applied chronologically. If a 28-day diary had 20 to 28 days of data, the measures of headache frequency and severity were prorated from the data recorded in the diary. If there were data for < 10 days in the diary, including for those who withdrew from the study, the number of headache days for the missing period was imputed by mLOCF based on the patient’s previous 28-day diary period and adjusted by the mean change observed in all patients with diary records for the same periods. The intent was to preserve the patient’s general position relative to the mean, using information from the patient and from the remaining patients. If there were data for 10 to 19 days, the number of headache days for the missing period was imputed by taking the average of the 2 estimates (the mLOCF estimate and the prorated estimate for the data recorded). For the secondary endpoint of HIT-6, for the baseline score if a patient answered < 50% of the questions on the HIT-6 survey, the HIT-6 score was set to missing; if ≥50% of the questions were answered, the total HIT-6 score was extrapolated from the mean score across all answered questions. For post-baseline visits, missing HIT-6 scores were imputed for all patients at each visit using mLOCF, based on the most recent results from a previous visit. For the subgroup analyses, missing headache days data and missing HIT-6 scores were imputed for all patients at each visit using a mLOCF.

Data from all investigative sites were pooled for the analyses. The analysis population included all enrolled patients who received ≥1 dose of onabotulinumtoxinA and ≥1 efficacy assessment. The safety population included all patients who received ≥1 dose of onabotulinumtoxinA.

A 2-sided paired *t* test was used to compare post-baseline efficacy outcomes with baseline efficacy outcomes, including testing the null hypothesis (that onabotulinumtoxinA treatment for 108 weeks caused no reduction in headache day frequency). A *P* value ≤0.05 was considered statistically significant. Similarly, a 2-sided group *t* test was used to assess differences between subgroups of two variables (ie, yes/no); one-way analysis of variance was used to test for differences between subgroups of three or more levels (eg, BMI).

## Results

### Patient disposition and demographics

A total of 716 patients were enrolled at 35 sites (United States, *n* = 572 [24 sites]; Korea, *n* = 80 [6 sites]; Australia, *n* = 64 [5 sites]). The intention-to-treat analysis population totaled 715 patients (United States, *n* = 571; Korea, *n* = 80; Australia, *n* = 64), and included 25 patients who reported < 15 headache days/month at baseline, despite having a diagnosis of CM. The safety population included all 716 enrolled patients who received ≥1 treatment with onabotulinumtoxinA.

**Fig. 2:**
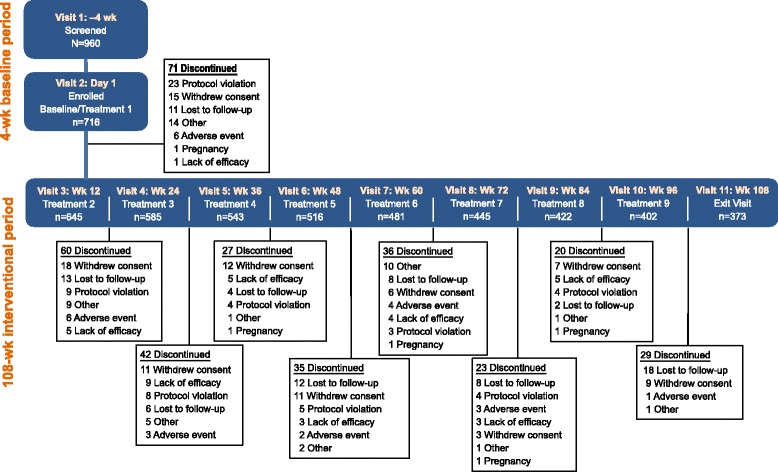
Patients who completed/withdrew from the study, including a summary of reasons for study withdrawal

All 9 study treatments were received by 402 patients (56.1%); 373 patients (52.1%) received all 9 study treatments and attended the final follow-up visit (ie, completed the study), and 343 patients (47.9%) withdrew from the study, primarily because of withdrawn consent (*n* = 92; 12.8%), being lost to follow-up (*n* = 82; 11.5%), or protocol violation (*n* = 60; 8.4%; Fig. 2). Suicidal ideation led to withdrawal from the study in 4 patients.

In the enrolled population, patients had a mean (SD) age of 43.0 (11.3) years and were predominantly female and Caucasian (Table 1). The mean (SD) age of onset of CM was 32.5 (13.7) years, patients had CM for a mean (SD) of 10.6 (11.0) years, and 62.7% of patients had a family history of migraine. Almost all patients (99.6%) reported headaches with moderate or severe pain and 68.2% of patients reported moderate or severe neck stiffness or pain.

**Table 1:** Baseline Demographics and Clinical Features of CM

Variable	Enrolled Population *N* = 716
Mean (SD) age, y	43.0 (11.3)
Female, n (%)	607 (84.8)
Race, n (%)	
Caucasian	582 (81.3)
Asian	89 (12.4)
African American/black	41 (5.7)
Other	4 (0.6)
Mean (SD) height, cm	165.8 (8.7)
Mean (SD) weight, kg	75.6 (19.8)
Mean (SD) BMI, kg/m^2^	27.4 (6.4)
Mean (SD) age of onset of CM, y	32.5 (13.7)
Mean (SD) time since onset of CM, y	10.6 (11.0)
Family history of migraine, yes, n (%)	449 (62.7)
Headache-related history,^a^ n (%)	
Sleep disorder	210 (29.3)
Smoking	150 (20.9)
Head trauma	74 (10.3)
Childhood abuse/maltreatment	50 (7.0)
Severity of pain during headache, n (%)	
Mild	3 (0.4)
Moderate	296 (41.3)
Severe	417 (58.2)
Pain on one or both sides of head, n (%)	
One	382 (53.4)
Both	334 (46.6)
Type of head pain, n (%)	
Throbbing or pulsing	507 (70.8)
Pressing or squeezing	170 (23.7)
Neither throbbing, pulsing, pressing, squeezing	39 (5.4)
Severity of neck pain or stiffness, n (%)	
Mild	70 (9.8)
Moderate	331 (46.2)
Severe	157 (21.9)
None	158 (22.1)
Other headache features, n (%)	
Sensitivity to light	658 (91.9)
Physical activity worsens headache	642 (89.7)
Sensitivity to noise	639 (89.2)
Nausea with headache	583 (81.4)
Vomiting with headache	295 (41.2)
Cutaneous allodynia	290 (40.5)
Mean (SD) headache days^b^	22.0 (4.8)
Mean (SD) moderate or severe headache days^b^	18.0 (5.7)
Mean (SD) HIT-6 total score^c^	64.7 (4.8)

*CM* chronic migraine, *HIT-6* 6-item Headache Impact Test

^a^Patients may be counted in > 1 category

^b^Headache days per 28 d in the analysis population (*n* = 715); includes 25 patients who reported < 15 headache days per 28 d at baseline

^c^In the analysis population (*n* = 715)

**Fig. 3:**
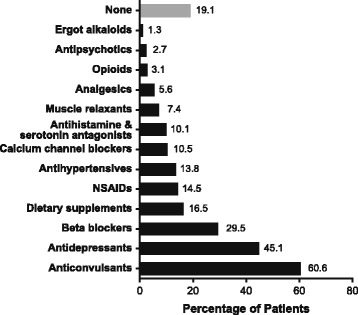
Oral preventive treatments* currently or previously used by the enrolled population (*N* = 716). *Oral preventive treatments defined, for the purposes of this study, as any oral medication *specifically* prescribed for *daily* use *for prevention of headache*. NSAID = nonsteroidal anti-inflammatory drug

At baseline, patients reported a mean (SD) of 22.0 (4.8) headache days per 28 days, with a mean (SD) of 18.0 (5.7) being moderate or severe. The majority of patients (89.2%) were using acute headache medications, and 63.7% were overusing their acute medication (Table 2). A total of 80.9% of patients had used oral preventive treatments in the past, with anticonvulsants (60.6%), antidepressants (45.1%), and beta blockers (29.5%) the most commonly used (Fig. 3). At baseline, 348 patients taking oral preventive treatments had a mean (SD) of 22.3 (4.8) headache days per 28 days; during the study 44 patients (6.1%) started taking an oral preventive treatments, most commonly topiramate (*n* = 18, 2.5%).

**Table 2:** Baseline Acute Headache Medication Use in Patients With CM^a^

Medication Use, n (%)	Enrolled Population *N* = 716
Acute headache medication use	639 (89.2)
Triptans	383 (53.5)
Simple analgesics	327 (45.7)
Combination analgesics	225 (31.4)
Opioids	117 (16.3)
Ergotamines	53 (7.4)
Acute headache medication overuse^b^	456 (63.7)
Triptans (≥10 d)	194 (27.1)
Combination analgesics (≥10 d)	88 (12.3)
Simple analgesics (≥15 d)	79 (11.0)
Opioids (≥10 d)	38 (5.3)
Ergotamines (≥10 d)	18 (2.5)

*CM* chronic migraine

^a^Data from patients with ≥20 d of data in their patient diary

^b^Definition of medication overuse is based on 4 wk. diary data

### Efficacy outcomes

**Fig. 4:**
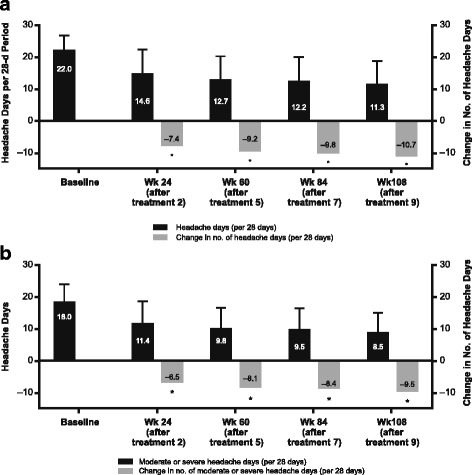
Long-term effect of onabotulinumtoxinA on number of and change vs baseline. **a**) Number of headache days and **b**) number of moderate or severe headache days per 28-d period preceding the visit over 108 wk. (depicting the outcomes of treatment after 9 cycles). *P < 0.0001; paired *t*-test used to compare visit to baseline

At week 108, onabotulinumtoxinA treatment reduced headache day frequency by 10.7 days from baseline, *P* < 0.0001 (Fig. 4a), with mean (SD) headache days reduced to 11.3 (7.4) days per 28-day period (from 22.0 [4.8] days at baseline; *P* < 0.0001).

Statistically significant reductions in headache day frequency were observed from the first assessment at week 24 (headache day frequency reduced by 7.4 days from baseline; *P* < 0.0001) and at all subsequent assessment points including week 60 (Fig. 4a). In a sensitivity analysis, similar reductions were observed at all time points using observed data without imputation (− 7.9 days at week 24 to − 11.6 days at week 108, all *P* < 0.0001). Based on observed data, the proportion of patients reporting a ≥ 50% reduction in headache days from baseline increased over the duration of the study, from 39.5% (223 of 565 patients) at week 24 to 61.1% (193 of 316 patients) at week 108.

**Fig. 5:**
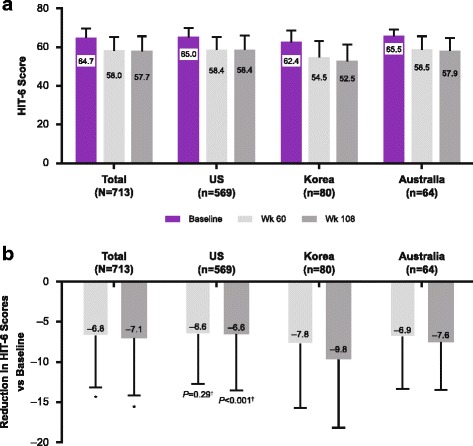
Long-term effect of onabotulinumtoxinA by country. **a**) HIT-6 total score and **b**) change in HIT-6 total score vs baseline, depicting the outcomes after 5 (wk 60) and 9 (wk 180) treatments. HIT-6 = 6-item Headache Impact Test. *Indicates *P* < 0.001 vs baseline; paired *t*-test used to compare visit to baseline. †Indicates *P* = 0.0008 for comparison between subgroups; 1-way analysis of variance

Baseline HIT-6 scores were available for 713 of 715 patients in the analysis population. Statistically significant improvements in total HIT-6 scores were observed at week 12 (HIT-6 score reduced by 4.4 points from baseline; *P* < 0.0001) and continued through to week 108 (− 7.1 from baseline; *P* < 0.0001; Fig. 5b). A similar pattern was seen using observed data (week 12: − 4.4 from baseline; week 108: − 9.0 from baseline; both *P* < 0.0001).

At baseline, patients had a mean (SD) of 18.0 (5.7) moderate or severe headache days. The frequency of moderate or severe headache days was reduced from baseline by 6.5 days at week 24, a statistically significant change from baseline (*P* < 0.0001; Fig. 4b). The reduction in moderate or severe headache days remained significantly reduced compared with baseline at week 60 (− 8.1 days; *P* < 0.0001) and at week 108 (− 9.5 days; *P* < 0.0001).

#### Subgroup analysis

No statistically significant between-group differences were observed for the change from baseline in the number of headache days at week 108 for the subgroups of race, comorbid depression, comorbid anxiety, history of acute headache medication overuse, age, or BMI (Additional file 1: Figures S1–S6). Statistically significant between-group differences were observed for preventive treatment at baseline versus no preventive treatment at baseline. Patients with preventive treatment at baseline had a significantly smaller reduction in mean (SD) headache days from baseline at week 108 than patients without preventive treatment at baseline (− 10.2 [6.3] vs − 11.2 [6.5]; *P* = 0.029, Additional file 1: Figure S7). Similar results were observed for the between group differences for previous use of preventive treatment versus no previous use of preventive treatment (Additional file 1: Figure S8).

**Fig. 6:**
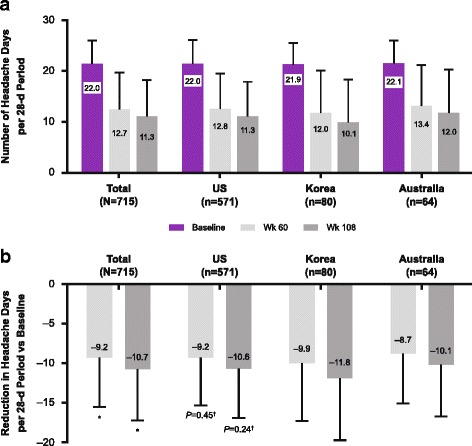
Long-term effect of onabotulinumtoxinA by country. **a**) Number of headache days per 28-d period and **b**) change in number of headache days vs baseline, depicting the outcomes after 5 (wk 60) and 9 (wk 180) treatments. *P < 0.001; paired *t*-test used to compare visit to baseline. †*P*-value for comparing among subgroups is from one-way analysis of variance

Similar reductions in headache day frequency were observed within each country subgroup; there were no statistically significant differences between countries (Fig. 6).

HIT-6 total score was significantly reduced from baseline in all subgroups at weeks 60 and 108. At week 108, the Korean subgroup had a significantly larger reduction in HIT-6 score compared with the US population (− 9.8 vs − 6.6; *P* < 0.001; Fig. 5b). In addition, non-Caucasian patients had a significantly greater reduction in HIT-6 score than Caucasian patients at week 108 (− 8.6 from baseline vs − 6.7 from baseline; *P* = 0.005; Additional file 1: Figure S1), as did the subgroup not on oral preventive treatment at baseline (− 7.4 from baseline vs − 6.1 from baseline for those on oral preventive treatment, at week 60 [*P* = 0.012]; − 8.0 vs − 6.1 at week 108 [*P* < 0.001]; Additional file 1: Figure S7). The outcomes for the subgroup on previous preventive treatment were almost identical (Additional file 1: Figure S8).

### Safety and tolerability

At least 1 treatment-emergent adverse event (TEAE) was reported by 436 patients (60.9%; Table 3). Of these, serious TEAEs occurred in 75 patients (10.5%). There were no deaths. TEAEs were reported in 32 patients (4.5%) who discontinued onabotulinumtoxinA. TEAEs that occurred in more than 2 patients who discontinued onabotulinumtoxinA were eyelid ptosis (*n* = 3, 0.4%), headache (*n* = 3, 0.4%), pregnancy (*n* = 3, 0.4%), suicidal ideation (*n* = 4, 0.6%) and rash (*n* = 3, 0.4%).

**Table 3:** Summary of TEAEs and TRAEs in Patients Receiving ≥1 OnabotulinumtoxinA Treatment

Event, n (%)	Safety Population *N* = 716
TEAE	
≥ 1 TEAE	436 (60.9)
Serious TEAE	75 (10.5)
TEAE in those that discontinued treatment	32 (4.5)
TRAE	
≥ 1 TRAE	131 (18.3)
Serious TRAE	1 (0.1)
TRAE in those that discontinued treatment	13 (1.8)
TRAE with incidence ≥1%	
Neck pain	29 (4.1)
Eyelid ptosis	18 (2.5)
Musculoskeletal stiffness	17 (2.4)
Injection site pain	14 (2.0)
Headache	12 (1.7)
Muscular weakness	10 (1.4)
Facial paresis	9 (1.3)
Migraine	7 (1.0)
Skin tightness	7 (1.0)

*TEAE* treatment-emergent adverse event, *TRAE* treatment-related adverse event

At least 1 treatment-related adverse event (TRAE) was reported in 131 patients (18.3%), with neck pain being the most commonly reported TRAE (Table 3). A TRAE was reported in 13 patients (1.8%) who discontinued onabotulinumtoxinA; with eyelid ptosis (*n* = 3, 0.4%) and rash (*n* = 3, 0.4%) being the most commonly reported TRAEs in patients who discontinued onabotulinumtoxinA. A serious TRAE of rash occurred in 1 patient (0.1%); onabotulinumtoxinA was discontinued in this patient.

## Discussion

The COMPEL Study provides clinical evidence for consistency of the efficacy and long-term safety and tolerability for onabotulinumtoxinA for the prevention of headache in those with CM who have been treated every 12 weeks over 2 years (9 treatment cycles) with the fixed-site, fixed-dose injection paradigm. OnabotulinumtoxinA effectively reduced headache day frequency compared with baseline over 9 treatment cycles (108 weeks), improved HIT-6 scores and reduced moderate or severe headache day frequency. These outcomes align with and further expand on the results of the double-blind, placebo-controlled phase of the PREEMPT trials over 24 weeks [22]. The baseline demographics of our population were similar to those of the group randomized to receive onabotulinumtoxinA in the first 24 weeks of the PREEMPT trials, except that our population had a shorter time since the onset of CM (10.6 years vs 19.2 years in the PREEMPT population) despite having a similar mean age (43.0 years vs 41.3 years). Therefore, our patients appeared to have a considerably older age at onset of CM than those in the PREEMPT population (32.5 vs 21.5; Allergan plc, data on file).

The reduction in headache day frequency from baseline after 24 weeks was similar to that observed in the PREEMPT trials (− 7.4 vs − 8.4); [22] as was the reduction in headache frequency after 5 treatments (week 60) in our study compared with those achieved after 5 treatments in the open-label phase of PREEMPT [17]. Notably after 56 weeks (5 treatments), onabotulinumtoxinA treatment reduced the mean headache day frequency by 11.7 days in the PREEMPT studies versus a mean reduction of 9.2 days at week 60 in our study.

PREEMPT allowed for a “follow the pain strategy” in addition to the base fixed-site fixed-dose treatment, allowing an additional 40 U of onabotulinumtoxinA to be administered at the clinician’s discretion, which could account for these slightly improved outcomes at week 56. In a population consisting only of those with medication overuse CM, onabotulinumtoxinA 195 U (administered according to the fixed-dose, fixed-site and the follow-the-pain injection paradigm) was similarly found to have significantly better outcomes than onabotulinumtoxinA 155 U (fixed-dose, fixed-site) over a 2-year period [23].

The optimal injection paradigm for onabotulinumtoxinA remains to be established. Simpler injection paradigms into fewer injection sites have also had successful outcomes; [24] most recently, onabotulinumtoxinA 70 U to 150 U injection into corrugator, temporalis, with or without the trapezius muscles, resulted in 72% of patients in a small (*N* = 63) real-life study experiencing ≥50% decrease in headache day frequency after ≥2 consecutive sets of injections [25]. Interestingly, small doses of onabotulinumtoxinA injected via acupoint sites (2.5 U per site, 25 U per treatment) was found to reduce migraine frequency, intensity, and duration by approximately 75% in Chinese patients (*N* = 102) with chronic migraine [26].

Our study allowed the addition or modification of oral preventive treatment at 24 weeks, which occurred in 44 patients (6.1%). This change in concomitant oral preventive treatment may have had an effect on efficacy outcomes. The efficacy outcomes may also be influenced by the inclusion of patients on oral preventive treatment at baseline. Patients on oral preventive treatment at baseline (*n* = 348) had a slightly smaller reduction in headache day frequency (− 10.2 from baseline vs − 11.2 from baseline, *P* = 0.029) and a statistically smaller reduction in HIT-6 total score at week 108 than patients not receiving oral preventive treatment at baseline (*n* = 367; − 6.1 from baseline vs − 8.0 from baseline, *P* < 0.001) despite having similar baseline headache day frequency (22.3 and 21.7 headache days per month, respectively) and HIT-6 scores (64.8 and 64.6, respectively). This suggests that those who were receiving oral preventive treatment at baseline may have had more refractory CM. Nonetheless, onabotulinumtoxinA still significantly reduced both headache day frequency (− 10.2 vs baseline, 95% CI –11.0 to − 9.5) and HIT-6 score (− 6.1 vs baseline, 95% CI –6.8 to − 5.4) at week 108 in this population versus baseline.

Our results further demonstrate that treatment with onabotulinumtoxinA through to week 108 provides continued improvement over 2 years, complementing previous observations in a smaller (*N* = 155) single-center study in patients with CM attributable to medication overuse [27]. In that study also the reduction in frequency of headache days was most pronounced after the first few treatment cycles and continued to further decrease through the 8th treatment cycle. Historically, it is recommended that after 12 months of good headache control, oral preventive treatment is reduced or discontinued; the goal being to establish the minimum effective dose [28]. In contrast, some public health bodies recommend that when onabotulinumtoxinA is used for the prevention of headaches it is stopped when the number of headache days per month drops below 15 days per month for 3 consecutive months [29]. Based on our study results and those of others, [27, 30] we question whether it is appropriate to discontinue onabotulinumtoxinA after only 3 months of remission to EM. The incremental benefits observed over the course of the COMPEL Study and that of PREEMPT [30] and Negro and colleagues [27] suggest that continuing treatment for up to 12 months, as recommended for oral preventive treatment, may be more beneficial to people with CM than early treatment withdrawal.

Clinicians report a 30% to 50% reduction in headache day frequency as a good response to treatment [29]. However, in our experience the majority of people with CM are seeking to maximize treatment response and maintain the improvement in their headaches. Combination of oral preventive treatment with long-term onabotulinumtoxinA treatment may be helpful in supporting people with CM to achieve this goal. The COMPEL Study is one of the first clinical studies of onabotulinumtoxinA in CM to allow the concomitant use of oral preventive treatment.

Management of those overusing headache medications is challenging; typically preventive treatments are ineffective during the period of acute medication overuse [28]. Public health bodies have asked for randomized controlled trials to investigate the role of appropriate pharmacological preventive treatment during acute medication withdrawal [31]. OnabotulinumtoxinA could be a useful treatment for such investigations as our results demonstrated it reduced headache day frequency and HIT-6 scores in individuals with medication overuse at baseline. Similarly, Negro and colleagues reported onabotulinumtoxinA 155 U and 195 U both resulted in significant reduction in headache days, HIT-6 scores and medication intake days in individuals with medication overuse CM, [23, 27] again suggesting an important role for onabotulinumtoxinA in this difficult to treat group.

Further analysis of the COMPEL Study will help describe the use of oral preventive treatment across regions and comorbidities and determine the impact of onabotulinumtoxinA on several factors including the impact on sleep, fatigue, and anxiety/depression, QoL and healthcare resource utilization.

The incidence of TEAEs and TRAEs in our study were similar to the incidence reported in the PREEMPT studies, [17] and in individuals with medication overuse CM, [23] further supporting the long-term safety and tolerability of onabotulinumtoxinA. As observed over the 56-week study period in the PREEMPT studies, neck pain (4.6%), eyelid ptosis (2.5%), muscular weakness (3.9%), injection site pain (2.0%), and muscle tightness (2.2%) were the most common TRAEs, closely matching the TRAEs reported over our 108-week study period. Our injectors were trained with insights gained from the PREEMPT Study, [32] supporting them to manage and avoid TRAEs such as eyelid ptosis and neck pain without reducing the dose or avoiding the injection site. Furthermore, by allowing the use of oral preventive treatment, the COMPEL Study data also provide some reassurance of the safety and tolerability of onabotulinumtoxinA in clinical practice. As the incidence of TEAEs and TRAEs in this current study were no worse than those reported in PREEMPT, the results of the COMPEL Study suggest that the addition of oral preventive treatment had no untoward impact on the safety profile of onabotulinumtoxinA.

As a large study in over 700 patients over 9 treatment cycles (108 weeks), the COMPEL Study has many strengths. In particular, the results help us to understand the ongoing efficacy and safety of onabotulinumtoxinA over prolonged treatment with the fixed-site and fixed-dose injection paradigm as well as in those taking concomitant oral preventive treatments and acute headache medications as they would in real-world conditions.

However, as a nonrandomized open-label study, our study is subject to inherent limitations particularly that there is no placebo or active comparator arm. An open label design is informative when the efficacy and safety profile of treatment is established, as it is with onabotulinumtoxinA for CM. However, open-label studies with long-term follow-up can be subject to unintentional bias, low persistency rates, and concomitant medication changes. Over a 2-year study, a relatively high level of discontinuation is to be expected; 56.1% (402/716) of patients received all 9 study treatments and 52.1% (373/716) received all study treatments and attended the final follow-up visit. The 2 primary reasons for discontinuation were withdrawn consent (12.8%) and lost to follow-up (11.5%). In the shorter PREEMPT studies, 72.6% of patients completed the 52-week study, with a lower proportion of patients (5.5%) lost to follow-up. Where patients referred to a single clinic for treatment of CM were enrolled in long-term studies of onabotulinumtoxinA, the withdrawal rates were substantially lower (14.8% and 16.9%), [23, 27] which is possibly more reflective of withdrawal rates in a real-world clinical setting.

Patients with no post baseline efficacy assessments were excluded from the analysis population; as only one patient was in this category, the impact on overall results would have been minimal. Because of the exclusion of patients with clinically significant conditions such as fibromyalgia, and those with severe depression and suicidal ideation, data from the patients who represent these or other populations who may be severely challenged may not have been captured [33]. Nonetheless, it is worth noting that that exploratory analyses of the COMPEL data demonstrated that concomitant mild or moderate anxiety or depression did not have a significant negative impact on outcomes. Efficacy results need to be interpreted with caution. The fixed-dose, fixed-site injection paradigm does not necessarily mirror real-world utilization and may underestimate real-world effectiveness, based on the slightly greater reduction in headache day frequency observed in the PREEMPT studies which included a follow-the-pain component to treatment. Conversely, the low persistency rates may overstate efficacy rates since those who do not experience benefit may not persist with treatment over the 108-week follow-up.

## Conclusions

The results of this international, multicenter, open-label, long-term prospective study support the efficacy and safety of onabotulinumtoxinA for prevention of headaches in adults with CM for up to 9 treatment cycles (108 weeks). Data indicated that onabotulinumtoxinA was effective in reducing headache days throughout 9 cycles of treatment, reducing the impact of headache from the first assessment (week 24). OnabotulinumtoxinA appeared to be well tolerated over 108 weeks and 9 cycles of treatment, and no new safety concerns were identified.

## Additional file

Additional file 1: Figure S1. Long-term effect of onabotulinumtoxinA on A) number of headache days per 28-d period, B)change in number of headache days vs baseline, C) HIT-6 score, and D) change in HIT-6 score vs baseline, depicting outcomes after 5 (wk 60) and 9 (wk 180) treatments, by race. **Figure S2.** Long-term effect of onabotulinumtoxinA on A) number of headache days per 28-d period, B) change in number of headache days, C) HIT-6 score, and D) change in HIT-6 score by comorbid anxiety group. **Figure S3.** Long-term effect of onabotulinumtoxinA on A) number of headache days per 28-d period, B) change in number of headache days, C) HIT-6 score, and D) change in HIT-6 score by comorbid depression group. **Figure S4.** Long-term effect of onabotulinumtoxinA on A) number of headache days per 28-d period, B) change in number of headache days, C) HIT-6 score, and D) change in HIT-6 score by BMI. **Figure S5.** Long-term effect of onabotulinumtoxinA on A) number of headache days per 28-d period, B) change in number of headache days, C) HIT-6 score, and D) change in HIT-6 score by history of acute medication overuse at baseline. **Figure S6.** Long-term effect of onabotulinumtoxinA on A) number of headache days per 28-d period, B) change in number of headache days, C) HIT-6 score, and D) change in HIT-6 score by age. **Figure S7.** Long-term effect of onabotulinumtoxinA on A) number of headache days per 28-d period, B) change in number of headache days, C) HIT-6 score, and D) change in HIT-6 score by use of oral preventive treatment at baseline. **Figure S8.** Long-term effect of onabotulinumtoxinA on A) number of headache days per 28-d period, B) change in number of headache days, C) HIT-6 score, and D) change in HIT-6 score by previous use of preventive treatment. (PDF 179 kb)

## Abbreviations

AEs: adverse events
BMI: body mass index
CM: chronic migraine
COMPEL: Chronic migraine OnabotulinuMtoxinA Prolonged Efficacy open Label
EM: episodic migraine
HIT-6: 6-item Headache Impact Test
IVRS: interactive voice response system
mLOCF: modified last observation carried forward
PREEMPT: Phase III REsearch Evaluating Migraine Prophylaxis Therapy
QoL: quality of life
SD: standard deviation
TEAE: treatment-emergent adverse event
TRAE: treatment-related adverse event
