# Supplementary material for: Long-term study of the efficacy and safety of OnabotulinumtoxinA for the prevention of chronic migraine: COMPEL study
Source: J Headache Pain. 2018 Feb 5;19(1):13. doi: 10.1186/s10194-018-0840-8 (PMC5799088; doi:10.1186/s10194-018-0840-8)
Supplement: Additional file 1: Figure S1. — Long-term effect of onabotulinumtoxinA on A) number of headache days per 28-d period, B)change in number of headache days vs baseline, C) HIT-6 score, and D) change in HIT-6 score vs baseline, depicting outcomes after 5 (wk 60) and 9 (wk 180) treatments, by race. Figure S2. Long-term effect of onabotulinumtoxinA on A) number of headache days per 28-d period, B) change in number of headache days, C) HIT-6 score, and D) change in HIT-6 score by comorbid anxiety group. Figure S3. Long-term effect of onabotulinumtoxinA on A) number of headache days per 28-d period, B) change in number of headache days, C) HIT-6 score, and D) change in HIT-6 score by comorbid depression group. Figure S4. Long-term effect of onabotulinumtoxinA on A) number of headache days per 28-d period, B) change in number of headache days, C) HIT-6 score, and D) change in HIT-6 score by BMI. Figure S5. Long-term effect of onabotulinumtoxinA on A) number of headache days per 28-d period, B) change in number of headache days, C) HIT-6 score, and D) change in HIT-6 score by history of acute medication overuse at baseline. Figure S6. Long-term effect of onabotulinumtoxinA on A) number of headache days per 28-d period, B) change in number of headache days, C) HIT-6 score, and D) change in HIT-6 score by age. Figure S7. Long-term effect of onabotulinumtoxinA on A) number of headache days per 28-d period, B) change in number of headache days, C) HIT-6 score, and D) change in HIT-6 score by use of oral preventive treatment at baseline. Figure S8. Long-term effect of onabotulinumtoxinA on A) number of headache days per 28-d period, B) change in number of headache days, C) HIT-6 score, and D) change in HIT-6 score by previous use of preventive treatment. (PDF 179 kb) [file 10194_2018_840_MOESM1_ESM.pdf]

Figure S1. Long-term effect of onabotulinumtoxinA on A) number of headache days per 28-d period, B) change in number of headache days vs baseline, C) HIT-6 score, and D) change in HIT-6 score vs baseline, depicting outcomes after 5 (wk 60) and 9 (wk 180) treatments, by race group.

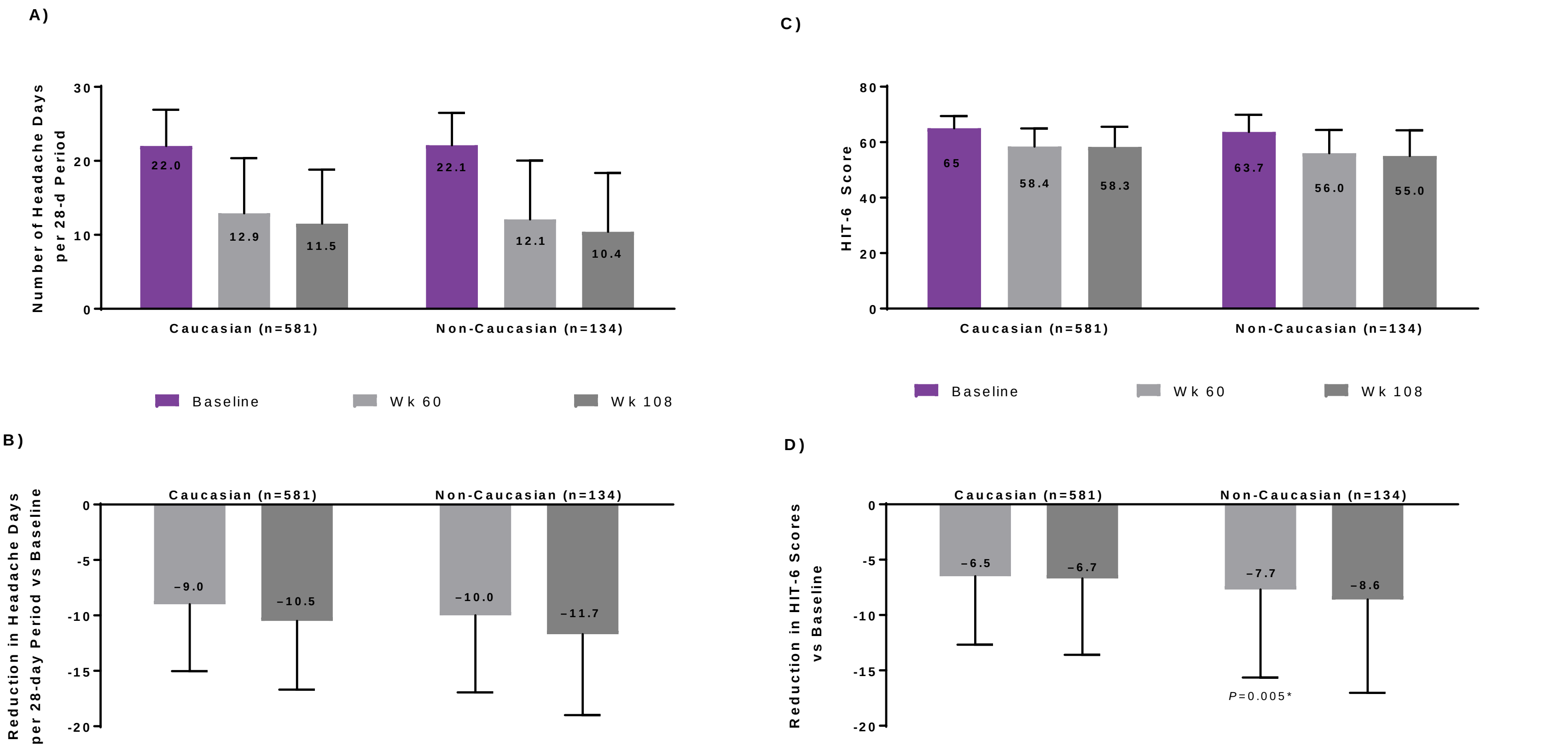

HIT-6=6-item Headache Impact Test.

*2-sided *P* value for comparing between subgroups is from the 2 independent groups *t*-tests.

Figure S2. Long-term effect of onabotulinumtoxinA on A) number of headache days per 28-d period, B) change in number of headache days vs baseline, C) HIT-6 score, and D) change in HIT-6 score vs baseline, depiciting outcomes after 5 (wk 60) and 9 (wk 180) treatments, by comorbid anxiety group.

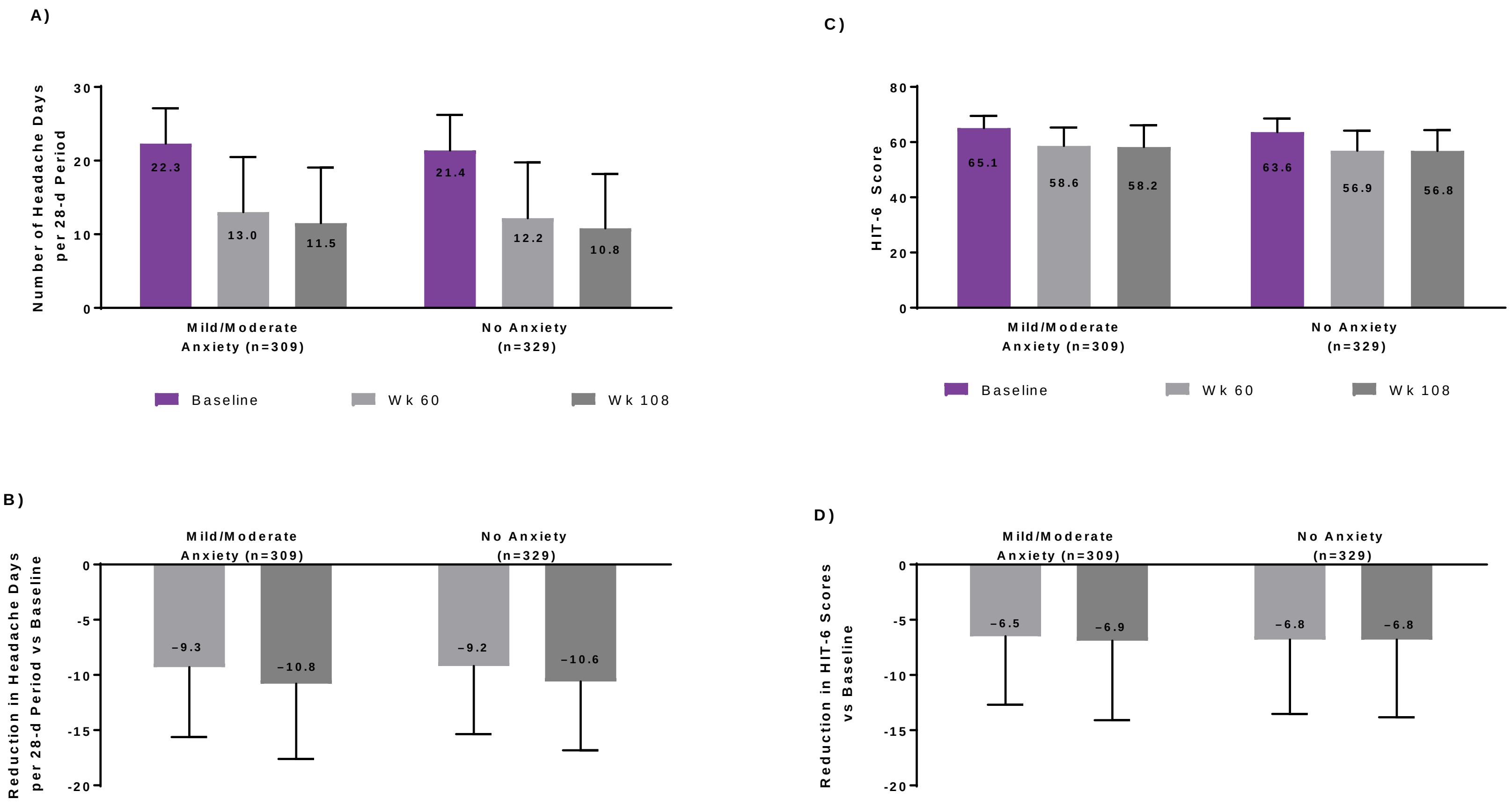

HIT-6=6-item Headache Impact Test.

Figure S3. Long-term effect of onabotulinumtoxinA on A) number of headache days per 28-d period, B) change in number of headache days vs baseline, C) HIT-6 score, and D) change in HIT-6 score vs baseline, depicting outcomes after 5 (wk 60) and 9 (wk 108) treatments, by comorbid depression group.

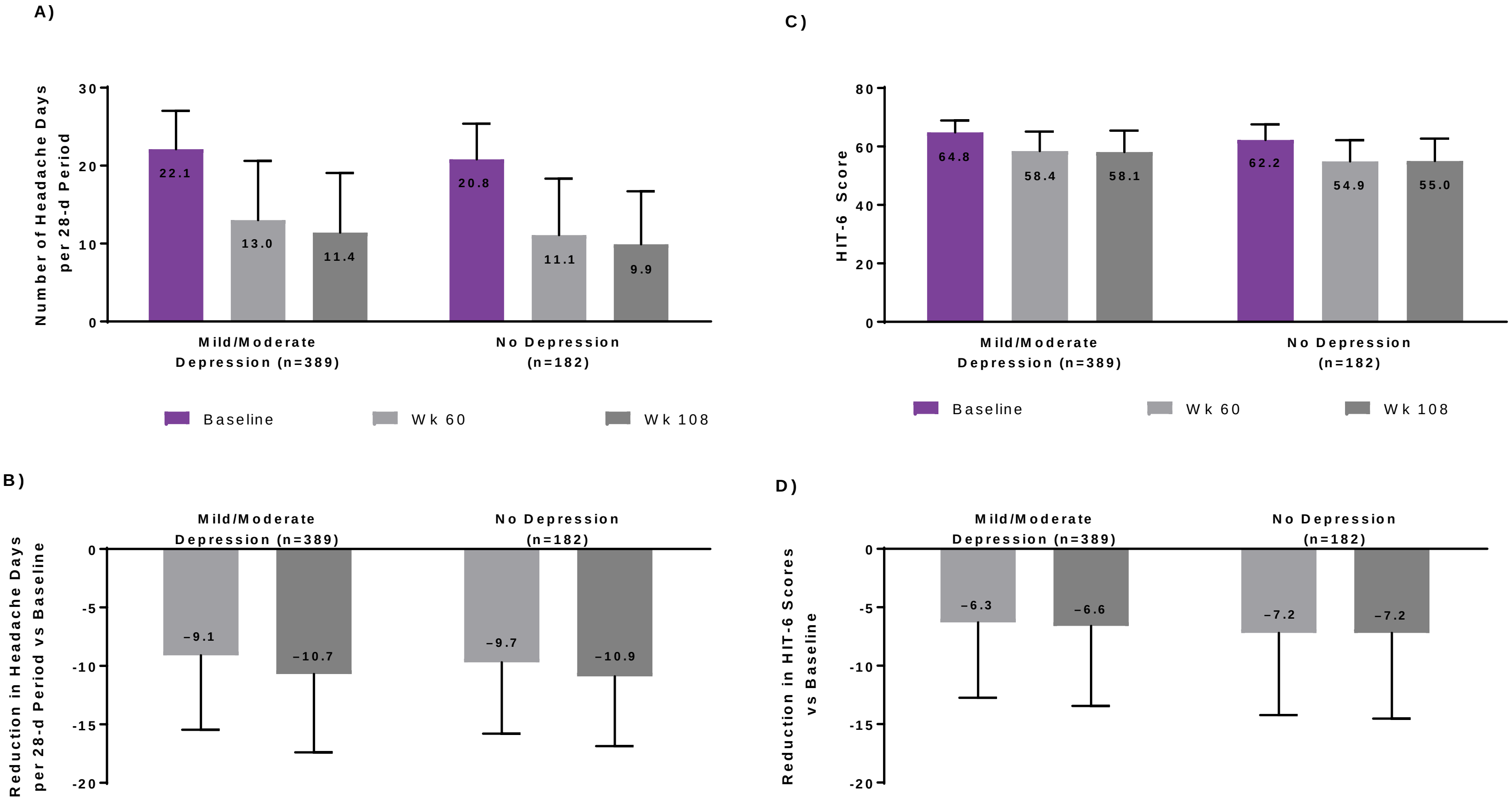

HIT-6=6-item Headache Impact Test.

Figure S4. Long-term effect of onabotulinumtoxinA on A) number of headache days per 28-d period, B) change in number of headache days vs baseline, C) HIT-6 score, and D) change in HIT-6 score vs baseline, depiciting outcomes after 5 (wk 60) and 9 (wk 180) treatments, by BMI.

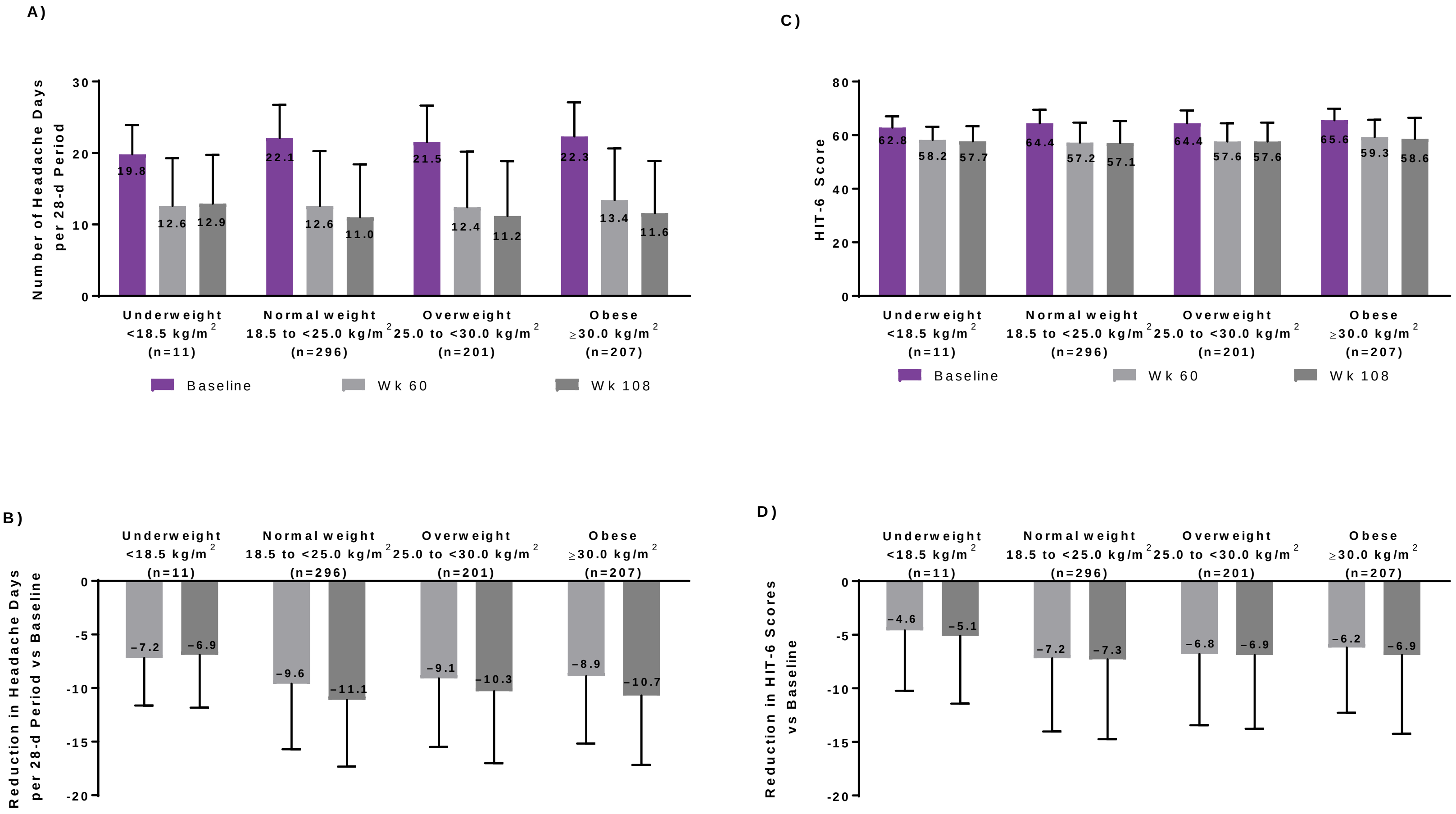

BMI=body mass index; HIT-6=6-item Headache Impact Test.

Figure S5. Long-term effect of onabotulinumtoxinA on A) number of headache days per 28-d period, B) change in number of headache days vs baseline, C) HIT-6 score, and D) change in HIT-6 score vs baseline, depicting outcomes after 5 (wk 60) and 9 (wk 108) treatments, by history of acute medication overuse at baseline.

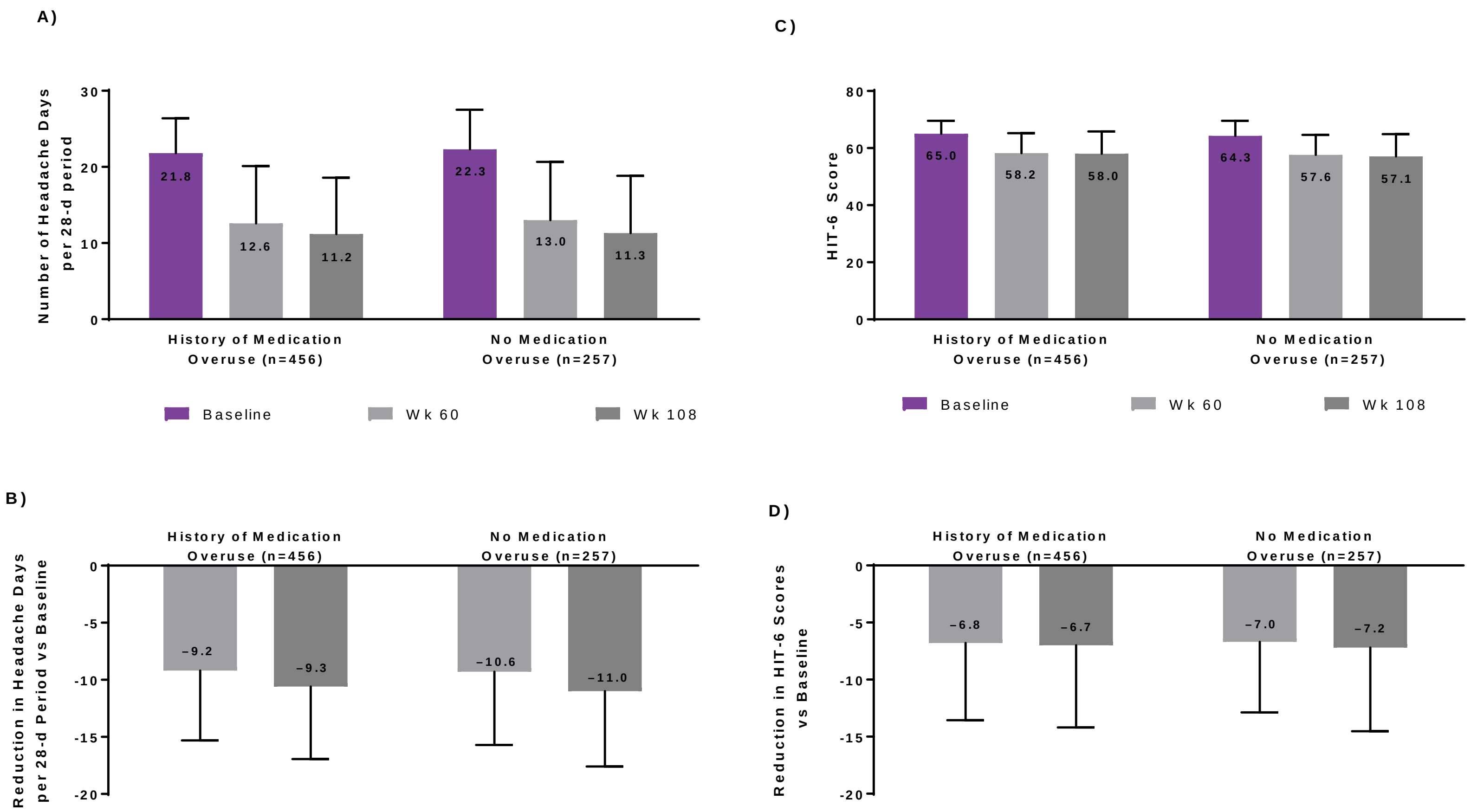

HIT-6=6-item Headache Impact Test.

*2-sided *P* value for comparing between subgroups is from the 2 independent groups *t*-tests.

Figure S6. Long-term effect of onabotulinumtoxinA on A) number of headache days per 28-d period, B) change in number of headache days vs baseline, C) HIT-6 score, and D) change in HIT-6 score vs baseline, depiciting outcomes after 5 (wk 60) and 9 (wk 180) treatments, by age.

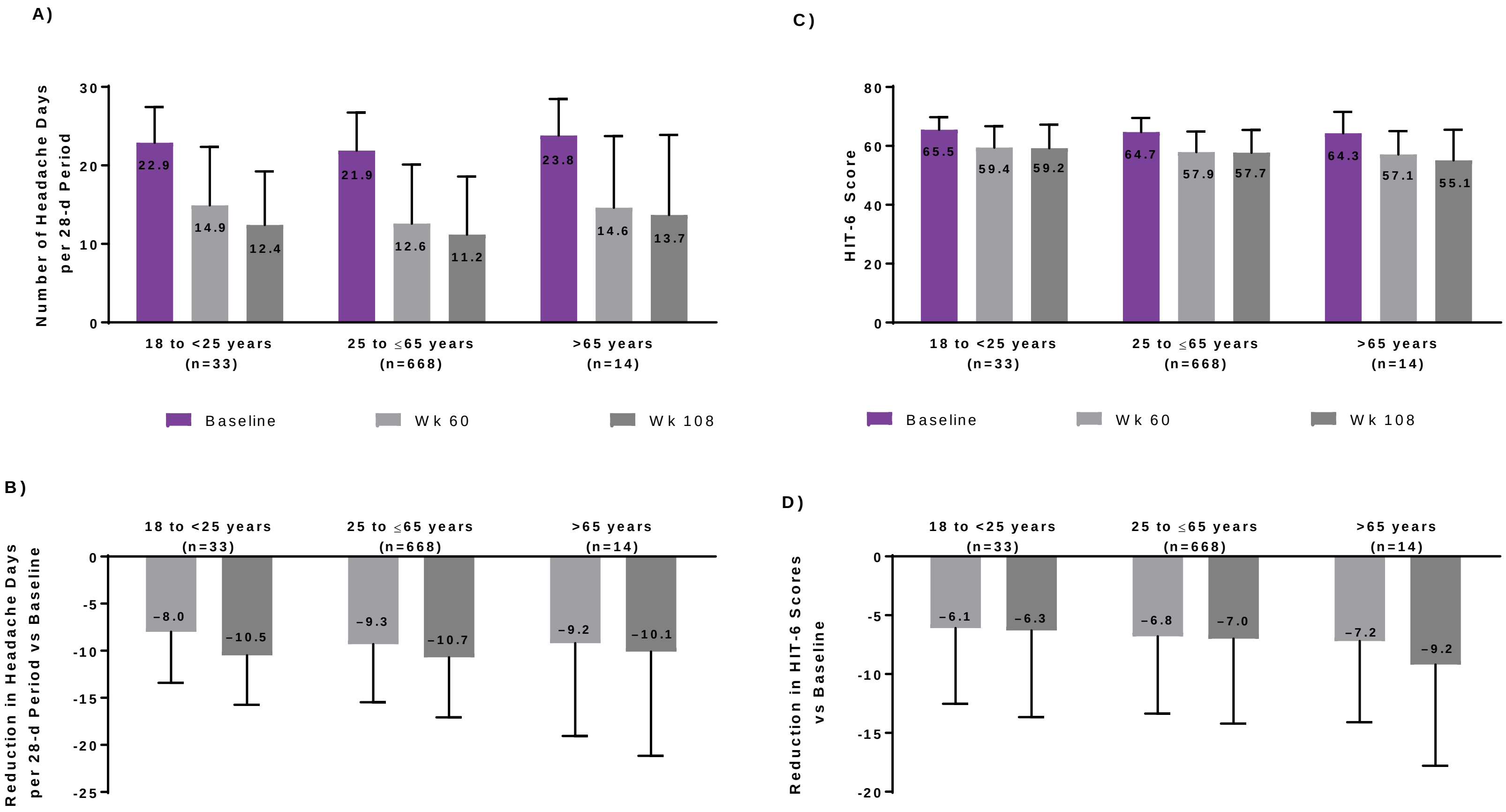

HIT-6=6-item Headache Impact Test.

Figure S7. Long-term effect of onabotulinumtoxinA on A) number of headache days per 28-d period, B) change in number of headache days vs baseline, C) HIT-6 score, and D) change in HIT-6 score vs baseline, depicting outcomes after 5 (wk 60) and 9 (wk 108) treatments, by use of oral preventive treatment at baseline.

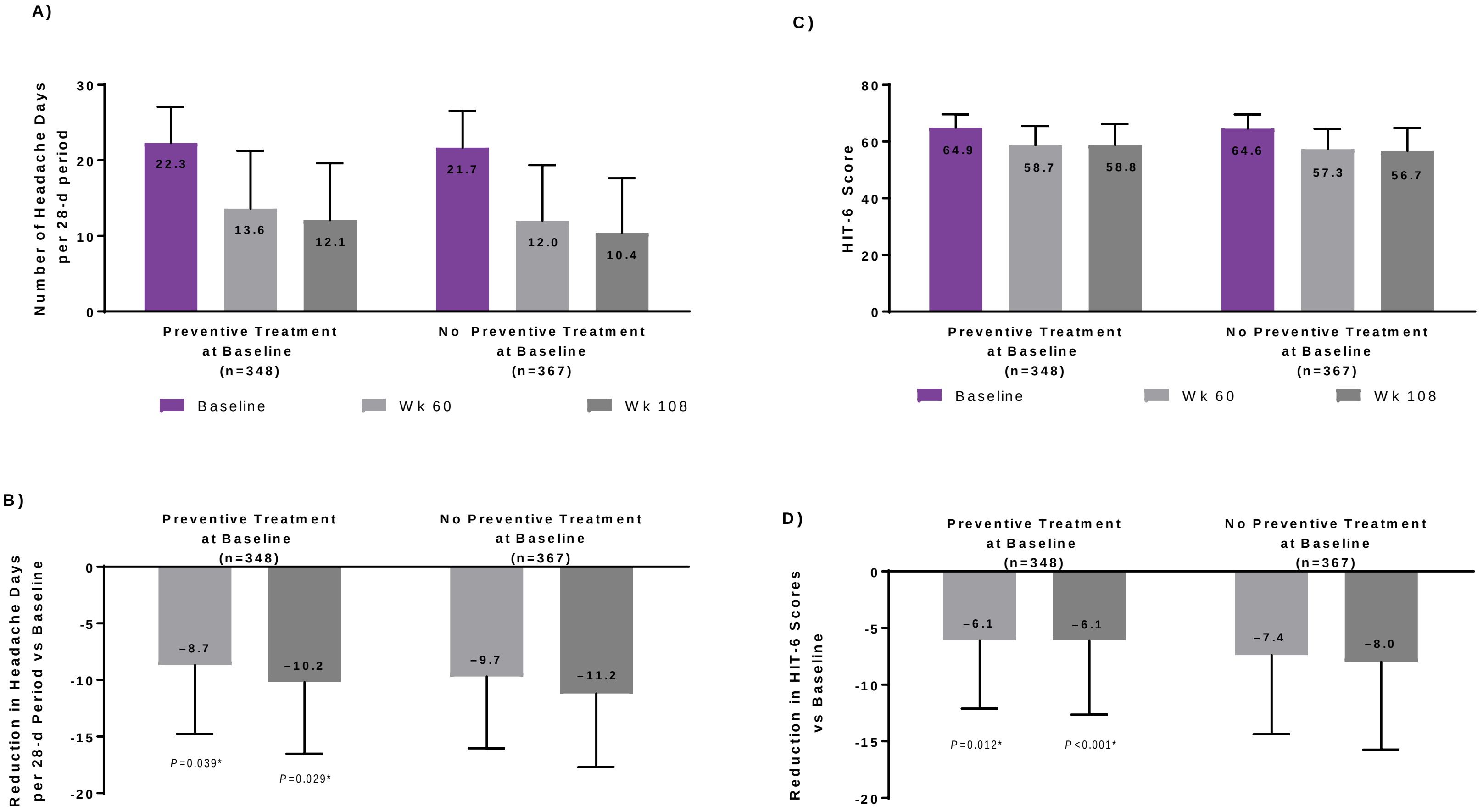

HIT-6=6-item Headache Impact Test.

*2-sided *P* value for comparing between subgroups is from the 2 independent groups *t*-tests.

Figure S8. Long-term effect of onabotulinumtoxinA on A) number of headache days per 28-d period, B) change in number of headache days vs baseline, C) HIT-6 score, and D) change in HIT-6 score vs baseline, depicting outcomes after 5 (wk 60) and 9 (wk 108) treatments, by previous use of preventive treatment.

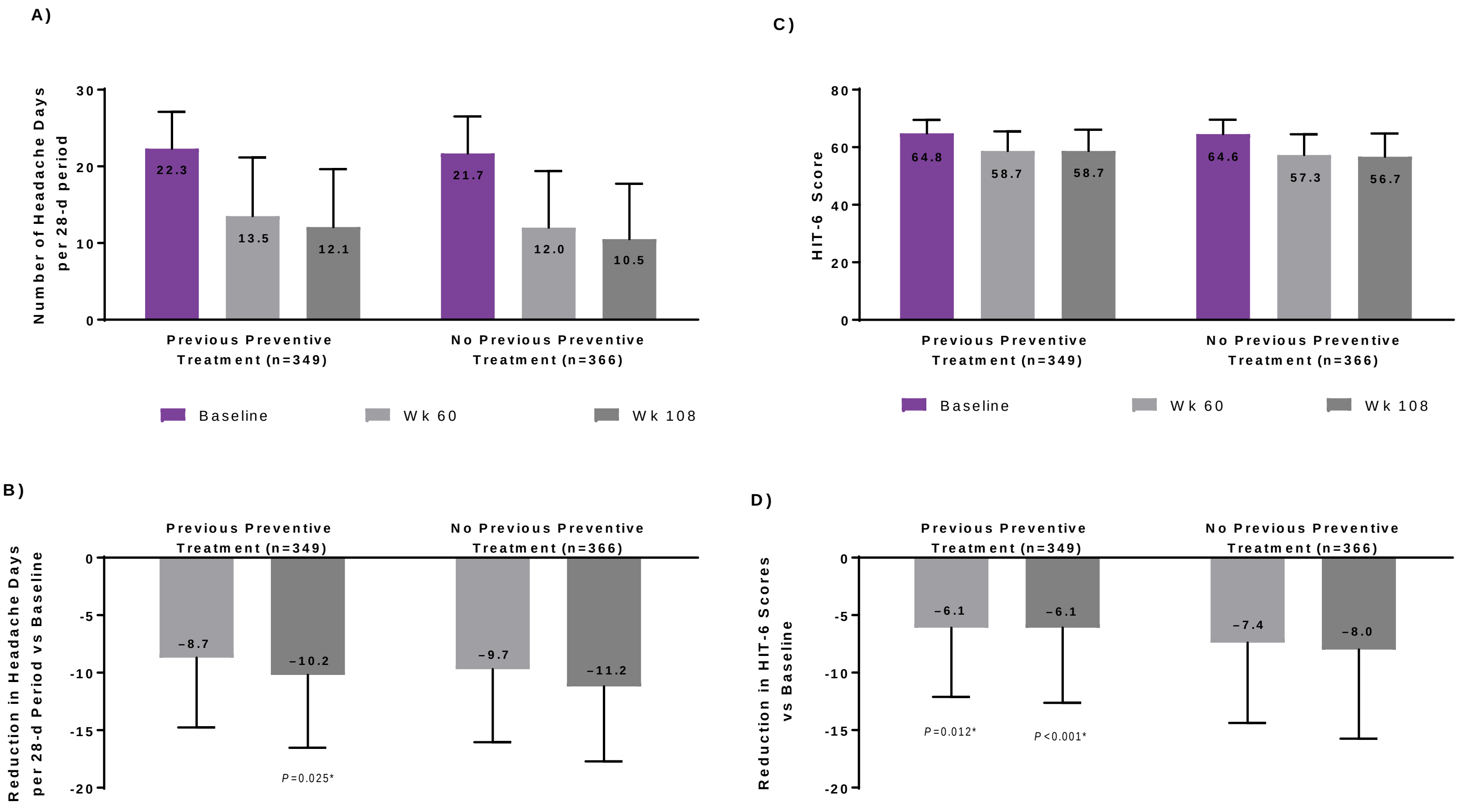

HIT-6=6-item Headache Impact Test.

*2-sided *P* value for comparing between subgroups is from the 2 independent groups *t*-tests.
